# Efficacy and factors influencing outcomes of customized music therapy combined with a follow-up system in chronic tinnitus patients

**DOI:** 10.1186/s40463-023-00631-y

**Published:** 2023-04-24

**Authors:** Yuehong Liu, Siyi Yang, Yulu Wang, Jiahua Hu, Hongbo Xie, Tianyi Ni, Zhao Han

**Affiliations:** Department of Otorhinolaryngology Head and Neck Surgery, Huadong Hospital Affiliated Fudan University, No. 221 West Yan’an Road, Jing An District, Shanghai, 200040 China

**Keywords:** Tinnitus, Customized music therapy, Efficacy, Influencing factor

## Abstract

**Backgrounds:**

Tinnitus is a meaningless sound signal perceived by the patients in the absence of auditory stimuli. Due to the complex etiology and unclear mechanism, specific therapies for tinnitus are still in the exploratory stage. In recent years, personalized and customized music therapy has been proposed as an effective method for tinnitus treatment. The aim of this study was to explore the efficacy of customized therapy with a well-designed follow-up system in the treatment of tinnitus through a large sample one arm study and to identify the relevant factors affecting the treatment outcome.

**Methods:**

The study investigated a total of 615 patients with unilateral or bilateral chronic tinnitus who received personalized and customized music therapy for 3 months. A complete follow-up system was designed by the professionals. Questionnaires of Tinnitus Handicap Inventory (THI), Hospital Anxiety and Depression Scale (HADS) and Visual Analogue Scale (VAS) were used to evaluate the therapeutic effects and relevant factors affecting the efficacy of therapy.

**Results:**

The results showed a decreasing trend in THI and VAS scores after 3 months of therapy, with statistically significant differences between pre- and post-therapy time points (*P* < 0.001). All patients were divided into 5 groups according to THI scores, and the mean reduction score in catastrophic, severe, moderate, mild and slight group was 28, 19, 11, 5, 0 respectively. The proportion of tinnitus patients with anxiety was higher than that with depression (70.57% and 40.65%, respectively), and there were statistically significant differences between HADS-A/D scores pre- and post-therapy. Binary logistic regression showed that the baseline of THI, VAS scores, the duration of tinnitus and the state of anxiety prior to therapy were significant influencing factors of therapeutic efficacy.

**Conclusions:**

The magnitude of reduction in THI scores after music therapy depended on the severity of the patients' tinnitus, the higher the initial THI scores, the greater the potential for improvement in tinnitus disorders. Music therapy also reduced the anxiety and depression levels of tinnitus patients. Therefore, personalized and customized music therapy with a comprehensive follow-up system may be an effective treatment option for chronic tinnitus patients.

**Graphical abstract:**

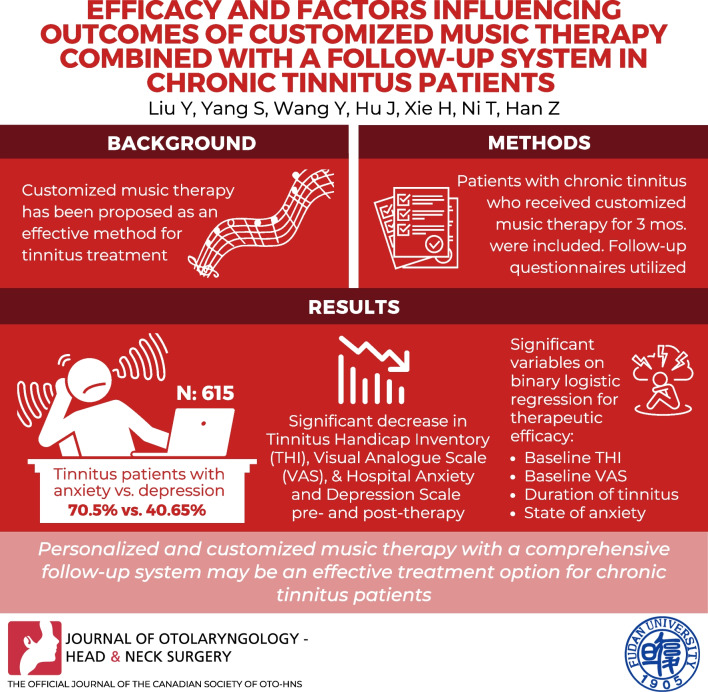

## Introduction

Tinnitus is one of the most common complaints in otorhinolaryngology and audiology outpatient clinic, and is usually classified as subjective and objective tinnitus. Epidemiological studies have shown that it can affect approximately 10–15% of adults [1, 2], leading to serious impact on quality of life [2–5].

The mechanisms underlying subjective tinnitus have not been fully elucidated. Based on current findings, the perception of tinnitus appears to emerge as an abnormal plasticity mechanism in cortical auditory system to compensate for reduced peripheral auditory input. Eggermont proposed three main mechanisms, named neural synchrony (hypersynchrony), central remodeling, and increased spontaneous discharge rate (hyperactivity) as neural correlates of tinnitus in the auditory system [6]. This uncertainty combined with related risk factors and associated comorbidities make tinnitus a difficult disorder to treat. In fact, a few treatments are designed to ameliorate the impacts of tinnitus rather than provide a cure for it [2], including medical and pharmacological intervention, sound therapy, and et al.

In recent years, based on the principle of central remodeling, Okamoto et al. proposed personalized treatment of subjective tinnitus using customized sounds to reduce tinnitus-related cortical activity and subjective tinnitus sensation [7]. Previous studies have shown that sound therapy can be effective in reducing the excitability of neural activity in the tinnitus brain [8]. Given this idea and the need for a convenient and clinically effective tinnitus treatment, a web-based, personalized and customized music therapy was applied in a large sample of tinnitus patients. In addition, a complete follow-up system was designed based on our previous studies [9] to solve the problem of not being able to follow up each patient individually due to the large sample size. We aimed to investigate the efficacy of the therapy for chronic, subjective tinnitus and to identify the factors that influence the treatment outcome.

## Materials and methods

### Participants

Patients with a chief compliant of tinnitus presenting to the otorhinolaryngology and audiology outpatient clinic were enrolled in this study. Inclusion criteria of subjective tinnitus included age ≥ 14 years, unilateral or bilateral subjective tinnitus lasting ≥ 1 year, with normal middle ear function, with or without sensorineural hearing loss, and access to a computer and participation in an internet-based trial. The reason why we selected 14 years of age as an entry criterion for patients is that our treatment relies on the internet and the age of 14 in China is basically an adult who can operate the mobile App on his or her own at the request of the doctor. Exclusion criteria included patients with ear disease, including otitis media, acoustic neuroma, and objective tinnitus, including middle ear myoclonus, vascular pulsatile tinnitus, as well as systemic diseases that may cause tinnitus symptoms, including hyperthyroidism, hypertension and diabetes, and tinnitus patients with cranial MRI suggestive of organic pathology, psychiatric disorders currently under therapy, communication disorders and currently receiving other sound or masking treatments.

### Evaluation methods of tinnitus

Tinnitus handicap inventory (THI), Hospital Anxiety and Depression Scale (HADS), and tinnitus loudness Visual Analog Scale (VAS) were used to describe the severity of tinnitus and to evaluate curative efficacy. THI is based on patient’s subjective perception of tinnitus, and consists of 25 items divided into functional, emotional, and catastrophic subscales that can be used to quantify the impact of tinnitus on daily life. THI scores range from 0 to 100 depending on the degree to which tinnitus affects the patients. According to the tinnitus severity scale proposed by McCombe et al. [10], patients were classified into 5 levels: slight (THI ≤ 16), mild (18 ≤ THI ≤ 36), moderate (38 ≤ THI ≤ 56), severe (58 ≤ THI ≤ 76), and catastrophic (THI ≥ 78). The higher the patient’s score, the more severe the effects of tinnitus. THI score is used to indicate the degree of negative effects [11]. The HADS consists of seven anxiety score items (HADS-A) and seven depression score items (HADS-D), each with a score of 0–21 points; the higher the score, the more severe the anxiety or depression symptoms [12]. "Loudness VAS" is used for evaluation the intensity of tinnitus on a scale ranging from 0 to 10 points. The higher the score, the louder the tinnitus. We compared the changes in HADS-A, HADS-D, THI, and VAS scores before and each month after treatment up to month 3 to assess the curative effects. Correlations between gender, age, frequency and duration of tinnitus, sides of tinnitus and outcomes of the evaluation indicators were also studied to identify factors affecting the efficacy of therapy.

### Interpretative consultation

Prior to treatment, counselling was provided in a face-to-face conversation of approximately 60 min using a standardized PowerPoint presentation. In the beginning, an introduction was provided of the auditory system, with special emphasis on the neural component. Then, a description of tinnitus mechanisms was given, including hypersyncrhony, hyperactivity, and reorganization of the tonotopic map, as compensatory response to deafferentation of auditory system. The comprehension of the neural connections between auditory and limbic systems helped to clarify the strong emotional responses to tinnitus, such as irritation, irritability, stress, anxiety, panic, and depression.

### Music production and therapy

Then, the production of customized music was accomplished by tinnitus assistant App’s core proprietary software (software that simulates tinnitus-related brain, provided by Sound Ocean Company and approved by the US FDA) with artificial intelligence. Firstly, the simulated tinnitus sound is obtained through 4 steps. The first step is to classify the characteristics of tinnitus in a given patient into 3 main categories namely common tinnitus sounds, electronic sounds, and normal daily life sounds. The second step is the secondary classification of the initial 3 categories of tinnitus sounds. Common tinnitus sounds include tonal/ringing, hissing/buzzing sounds or insect sounds; electronic sounds include different types of motor/mechanic sounds; and normal daily life sounds include television snowflake and kettle boiling sounds. The third step is to match the patient-specific frequency classification based on the previously selected type of tinnitus. The final step is to match the patient’s tinnitus loudness based on the different volume levels resolved by the program from 1 to 10 degree. Then the simulated tinnitus sound is input into the simulated brain (tinnitus assistant App), and the active areas of the response are observed and recorded by a computer to simulate the brain response. Then the frequency, intensity and phase of the music that is in the range of the patient's hearing-impaired frequencies are edited and adjusted by inverse-phase editing procedure, so that the frequency and intensity of the music are the same as the simulated tinnitus sound but with an opposite phase. According to the superposition property of waves, superposition of the music and the tinnitus sound is able to eliminate the latter. Therefore, tinnitus sound can be neutralized by the music; while if the patient is free of tinnitus, the music can stimulate the patient's brain in the opposite direction, reducing the sensitivity of the tinnitus sound and achieving the purpose of rehabilitation.

The patients were instructed to use the recommended headphones and receive personalized music therapy in a quiet environment without outside sound interference for a total of at least 2 h per day. The loudness of therapeutic sound was kept at the same level as the loudness of tinnitus, to which degree tinnitus sound can be heard by the patients while concentrating on therapeutic music. For those patients with fluctuating loudness or changes in the type of tinnitus, they can contact the relevant professional for professional guidance through the App.

### Follow up system

All patients were followed up to the third month by the tinnitus assistant App, which will automatically record the therapy time and supervise the patient to complete the time as required. Meanwhile, the follow-up and counseling systems have been installed under professional guidance. Patients were assessed electronically for AHI, VAS and HADS on a monthly basis and every patient was asked to complete and submit all the questionnaires. Back-and-forth communications about treatment-related problems were replied and patients were urged to listen to the customized sounds as required. Improprieties in the treatment process would be pointed out and corrected in time.

## Results

### Participants characteristics

In our study, there were altogether 615 outpatients who met the inclusion criteria (see Table 1 for details), with tinnitus as the main complaints. There were 309 females and 306 males. Their ages ranged from 14 to 80 years (mean ± SD, 41.62 ± 12.52 years). The mean duration of tinnitus before therapy was 4.01 ± 8.28 years. Other pre-therapy characteristics were shown in Table 1. Notably, more tinnitus patients suffered from anxiety than depression (70.57% vs. 40.65%). The prevalence of anxiety or depression was as high as 75.28%, suggesting a strong link between tinnitus and emotional disorders. All the patients were divided into 5 different distress groups (including catastrophic, severe, moderate, mild and slight). The results showed that 29 (4.7%) patients who scored less than 18 points had slight disturbance, 17.2% of the participants had mild disturbance, 30.7% suffered from moderate disturbance, 23.7% were concerned by severe disturbance, and 23.6% experienced catastrophic disturbance, with the moderate group accounted for the highest number of patients. Moreover, we found that a few patients with low THI scores and high HADS-A/D scores in the slight group sought for treatment, which suggests that tinnitus disability is not an absolute factor bothering patients, and that emotional factors may also be an important reason for patients to seek medical help.

**Table 1:** Pre-thearapy characteristics of patients

Pre-therapy characteristics	
Characteristics	Sum *n* = 615
Sex (M:F)	306:309
Age (year)	41.62 ± 12.52 (14 ~ 80)
Tinnitus duration (year)	4.01 ± 8.28
Unilateral:bilateral	267:348
*Tinnitus severity levels*	
Catastrophic(78–100)	145 (23.6%)
Severe(58–76)	146 (23.7%)
Moderate(38–56)	189 (30.7%)
Mild(18–36)	106 (17.2%)
Slight(0–16)	29 (4.7%)
Anxiety proportion(A ≥ 8)	434 (70.57%)
Depression proportion(D ≥ 8)	250 (40.65%)
Anxiety or depression proportion (A&D ≥ 8)	463 (75.28%)

The patients receiving music therapy were well informed and educated prior to implementation.

### Tinnitus severity levels are correlated with depression/anxiety levels and VAS scores

Spearman correlation analysis was used to examine the relationships between tinnitus severity and depression/anxiety levels and VAS scores. Results based on Table 2 showed that THI score was positively correlated with depression/anxiety/VAS scores prior to therapy (*p* < 0.01, *n* = 615) as well as 3 months post-therapy (*p* < 0.01, *n* = 593). The correlation between THI and HADS-A was somewhat stronger than that between THI and HADS-D (r = 0.620 vs. r = 0.542 pre-therapy and r = 0.641 vs. r = 0.490 post-therapy). However, the correlation between VAS and HADS-A/HADS-D was relatively poor (r = 0.403 & 0.365 pre-therapy and r = 0.418 & 0.376 post-therapy).

**Table 2:** Correlations between tinnitus severity and depression/anxiety/VAS levels

Pre-therapy	THI	HADS-A	HADS-D	VAS
THI	1	0.620**	0.542**	0.590**
HADS-A	–	1	0.565**	0.403**
HADS-D	–	–	1	0.365**
VAS	–	–	–	1

3 M-post-therapy	THI	HADS-A	HADS-D	VAS
THI	1	0.641**	0.490**	0.626**
HADS-A	–	1	0.561**	0.418**
HADS-D	–	–	1	0.376**
VAS	–	–	–	1

**Significant correlation at the 0.01 level (bilateral), Spearman correlation analysis; *THI* Tinnitus Handicap Inventory; *HADS* Hospital Anxiety and Depression Scale; *A* Anxiety;D:Depression; *VAS* Visual Analogue Scale

### Curative effect of music therapy with follow-up system for subjective tinnitus

The results of the study showed a decreasing trend in THI, HADS and VAS scores (Table 3). A total of 589 patients in the study participated in the therapy and completed all the THI scores, in which the median THI decreased from 56 (38,74) to 40 (18,55) with a reduction of 16 points. Statistically significant differences were found between the values obtained at pre-therapy and post-therapy time points (*P* < 0.001). Also, there were statistically significant differences between values obtained 1 month post-therapy and 2 or 3 months post-therapy (*P* < 0.05), but not between 2 and 3 months post-therapy (*P* > 0.05).

**Table 3:** Treatment outcomes after 3 months of personalized, customized music therapy

Therapy time	Baseline	1 month	2 months	3 months	P*
THI	56 (38,74)	44 (26, 62)^a^	40 (24,58)^a,b^	40 (18,55)^a,b^	< 0.001
HADS-A	9 (7,12)	8 (6,10)^a^	8 (5,10)^a^	7 (5,9)^a,b^	< 0.001
HADS-D	6 (4,10)	6 (3,9)^a^	6 (3,9)^a^	5 (2,9)^a,b^	< 0.001
VAS	6 (4,7)	5 (3,6)^a^	4 (3,6)^a,b^	4 (3,6)^a,b^	< 0.001

* = Friedman test; Superscript letters indicate

a = comparison with baseline (*P* < 0.001)

b = comparison with 1 month after therapy (*P* < 0.05); data are presented as Median (lower quartile, upper quartile); *THI* Tinnitus Handicap Inventory; *HADS* Hospital Anxiety and Depression Scale; *A* Anxiety;D:Depression; *VAS* Visual Analogue Scale

**Fig. 1:**
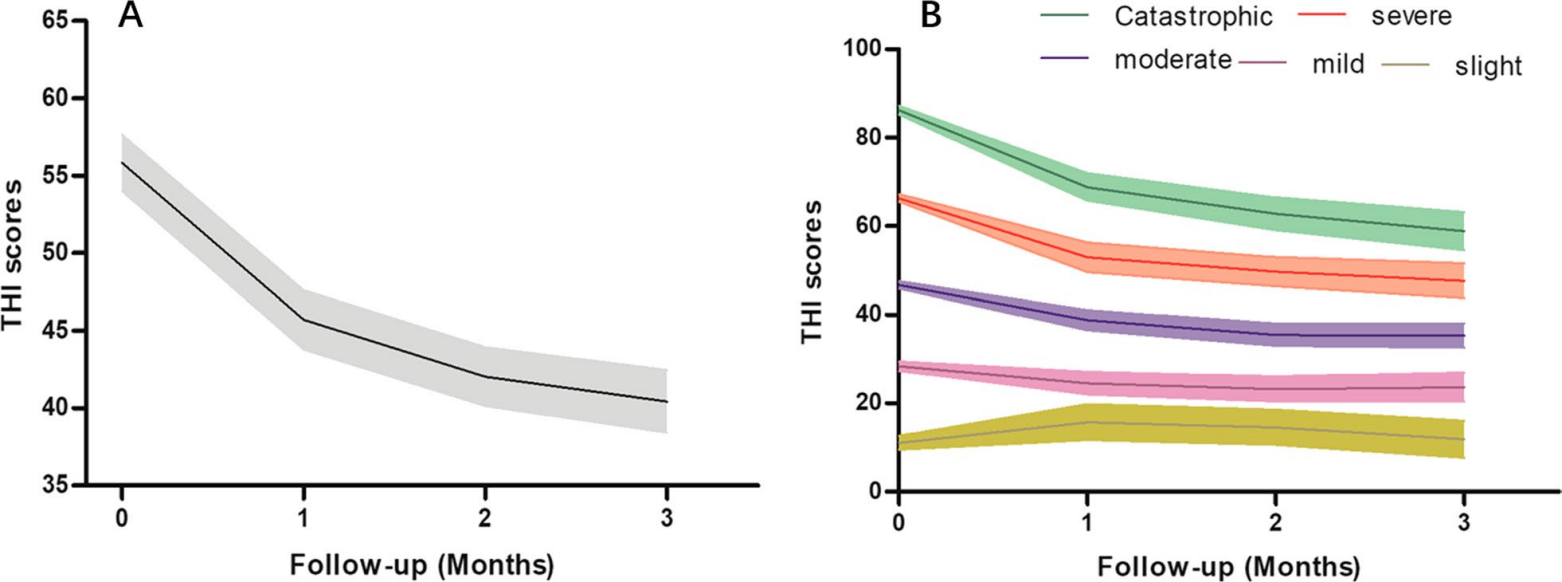
**A** The overall average THI score changes over time. **B** The average THI score changes over time for the different distress groups. Shaded areas around the mean THI display the 95% confidence intervals

Figure 1 showed the tinnitus distress reduction performed by severity levels, which implied that the more severe the THI grouping level, the more obvious the drop in the curve. In the mild group, however, there was a slight increase after 1 month of treatment. The results of this analysis are summarized in Table 4. Which showed that there was no change in mean THI scores for patients in the slight group. For the mild and moderate group, the mean THI score was reduced by 5 and 11 points, respectively. However, for the severe and catastrophic group, greater reductions of 19 and 28 points were obtained post-therapy. We therefore confirm that the final THI reduction post-therapy increases with increasing baseline THI values.

**Table 4:** Average THI conduction by severity levels

Severity Scale	THI_ini_	THI_fin_	∆THI
Slight	11	11	0
Mild	28	23	− 5
Moderate	46	35	− 11
Severe	66	47	− 19
Catastrophic	86	58	− 28
Overall	56	40	− 16

*THI* Tinnitus Handicap Inventory

**Fig. 2:**
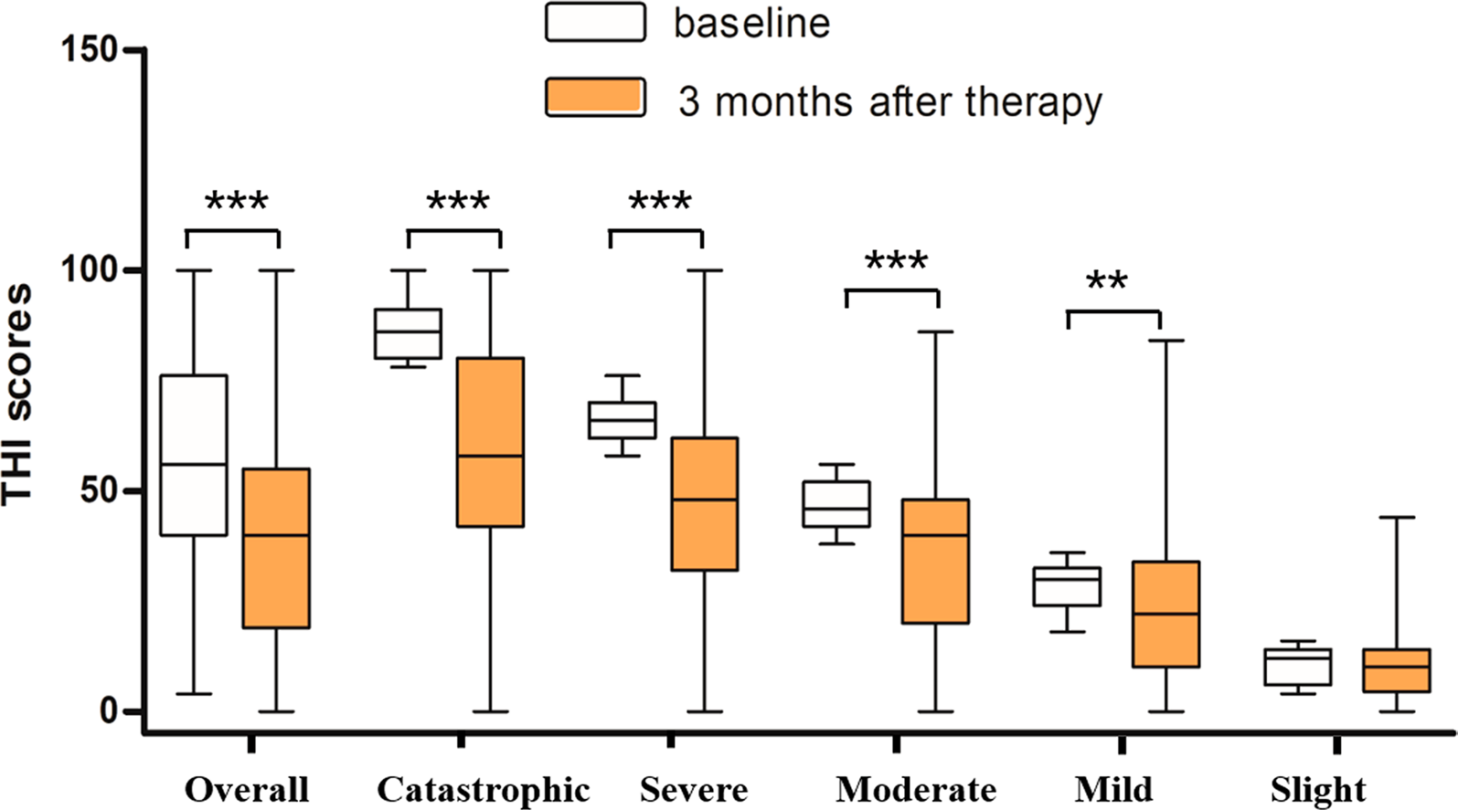
Median THI scores before and after treatment for the different distress groups. * = Wilcoxon signed-rank test *** represents statisitical significance at *P* < 0.001; ** represents statisitical significance at *P* < 0.05

Figure 2 showed a box plot of the THI score reduction by severity. It showed that there was a statistically significant difference between the scores before and after therapy for the catastrophic, severe, moderate and mild groups. While for the slight group, no significant difference was obtained. As shown in Table 5, the percentage of catastrophic group was 23.6% before therapy, which dropped to 11.4% 1 month after therapy and then to 10.8% 3 months after therapy. While for the severe group, the percentage decreased from the initial 23.7% before therapy to 12.3% after 3 months’s therapy. In contrast, the percentage of patients in the mild group tended to increase, from 17.2% of the baseline to 22.9% after 3 months of therapy. Meanwhile, patients in the slight group showed the greatest change, rising from 4.7% pre-therapy to 21.4% post-therapy. The fact that THI scores in the slight group were present in normal subjects means that after 3 months of treatment, part of tinnitus patients had achieved clinical cure.

**Table 5:** Change in percentage of each distress group before and after therapy

Severity levels	Catastrophic (78–100)	Severe (58–76)	Moderate (38–56)	Mild (18–36)	Slight (0–16)
*Therapy time*					
Baseline (*n* = 615)	145 (23.6%)	146 (23.7%)	189 (30.7%)	106 (17.2%)	29 (4.7%)
1 M (*n* = 606)^a^	69 (11.4%)	124 (20.5%)	190 (31.4%)	140 (23.1%)	83 (13.7%)
3 M (*n* = 593)^a,b^	64 (10.8%)	73 (12.3%)	193 (32.5%)	136 (22.9%)	127 (21.4%)

All the data are presented as: number of cases (% share). Superscript letters indicate: a = comparison with baseline (*P* < 0.001); b = comparison with 1 month after therapy (*P* < 0.01); *P* = Wilcoxon related samples test

According to the VAS results in Table 3, the median VAS score before therapy was 6, decreasing to 5 after 1 month of therapy and finally to 4 after 3 months of therapy. There were statistically significant differences between the values obtained at the pre- and post-therapy time points. There were also statistically significant differences between values obtained 1 month after therapy and 2 or 3 months after therapy, but no differences between 2 and 3 months after therapy.

### Music therapy could reduce anxiety and depression levels in tinnitus patients

Of the 615 patients rolled in this study, 70.57% (434/615) of them suffered from anxiety disorders and 40.65(250/615) suffered from depression. After 3 months of therapy, HADS scores were obtained from 418 anxious patients and 239 depressed patients who were still being followed up. The results showed that 44.98% (188/418) of the anxious patients reported anxiety scores within negative (no anxiety) range (A < 8) and 46.03% (110/239) of the depressed patients reported depression scores within negative (no depression) range (A < 8). If a decrease of one level in HADS-A/D scores was considered effective for the therapy (0–7: asymptomatic; 8–10: suspicious; 11–21: definite present), for instance, from suspicious group to asymptomatic group, the treatment effectiveness rates were 61.72% (258/418) and 61.09% (146/239) for anxiety and depression, respectively.

According to Table 3, the median value of the anxiety scores before therapy was 9, which decreased to 8 after 1 month of therapy and to 7 after 3 months of therapy. Statistical differences were found between values obtained pre- and post-therapy time points, and between 1 and 3 months after therapy, with no statistical differences between other time points. The median value of the depression score prior to therapy was 6, which decreased to 5 after 3 months of therapy. Similarly, there were statistically significant differences between the pre- and post-therapy time points, and between 1 and 3 months post-therapy, while no statistically significant differences were found between the other time points.

### Factors influencing the efficacy of music therapy

To further explore the factors influencing the efficacy of music therapy, further analysis was conducted using binary logistic regression. As shown in Table 6. the frequency and left–right laterality of tinnitus, age, gender, and the presence of depression prior to therapy had no effect on the efficacy of music therapy (*P* > 0.05). In contrast, the severity of tinnitus at baseline and VAS levels were significantly influencing factors. The more severe the tinnitus, the better the outcome (*P* < 0.001). Duration of tinnitus also influenced the efficacy, with longer duration of tinnitus associated with poorer outcome (*P* < 0.05). The efficacy of patients with tinnitus lasting less than 3 years was 1.762 times higher than that of patients with tinnitus lasting more than 3 years. Notably, we found that whether patients were in a state of anxiety prior to therapy also affected the effectiveness of customized music therapy (*P* < 0.05). The efficacy of the therapy was 1.977 times higher in tinnitus patients with pre-therapy anxiety than in those without anxiety.

**Table 6:** Analysis of factors influencing the effect after 3 months of music therapy

Factors	B	SE	Wald	*P*	OR	95% CI
Baseline	1.263	0.239	28.040	< 0.001	3.537	2.216–5.645
(THI ≥ 58 vs. < 58)						
Age	− 0.021	0.196	0.011	0.916	0.980	0.667–1.438
(> 40 years vs. ≤ 40 years)						
Sex (M vs. F)	0.283	0.195	2.121	0.145	1.328	0.907–1.944
Frequency	0.348	0.228	2.333	0.127	1.417	0.906–2.215
(> 4 kHz vs. ≤ 4 kHz)						
Duration	0.566	0.244	5.372	0.020	1.762	1.091–2.844
(≤ 3 years vs. > 3 years)						
Sides	0.228	0.195	1.362	0.243	1.256	0.857–1.841
(unilateral vs. bilateral)						
Anxiety	0.682	0.224	9.247	0.002	1.977	1.274–3.068
(with vs. without)						
Depression	− 0.027	0.215	0.016	0.901	0.974	0.638–1.484
(with vs. without)						
VAS scores	0.485	0.244	3.958	0.047	1.624	1.007–2.617
(≤ 6 vs. > 6)						

Efficacy evaluation of therapy: THI score drops by one level or more; *THI* Tinnitus Handicap Inventory; *VAS* Visual Analogue Scale

### Analysis of the lost to follow (LTF)

After 1 month of therapy, 9 patients were lost to follow-up (LTF = 1.46%) and after 3 months of therapy, 22 patients were lost to follow-up (LTF = 3.58%). 12 patients stopped therapy on their own because the tinnitus improved or disappeared after 1–3 months of treatment; 7 patients lost to follow because they forgot to do the questionnaires and 3 stopped because they felt that the tinnitus worsened.

## Discussion

Tinnitus affects the patient’s daily life, interferes with concentration and sleep quality, which hinders mental work, causes anxiety and depression, and in the extreme, makes some patients prone to suicide [13]. Therefore, it is an urgent problem for otologists to address.

Assessment of tinnitus was committed through application of two internationally recognized evaluation instruments, the THI and the VAS. Tinnitus associated emotional disorders were assessed by applying the Chinese version of HADS. THI has been widely used worldwide since its development in 1996 [14]. The psychometric properties of the THI have been validated in many different versions, including the Chinese version [15]. The VAS was commonly used to assess pain level [16], but has also been proved to assess inner ear sound loudness in patients with tinnitus [17]. Using the VAS together with THI may be useful to assess subjective levels of tinnitus-specific psychoacoustic features (e.g., loudness) not covered by the THI as continuous variables (relative to yes or no responses). Thus, previous studies on tinnitus have applied both THI and VAS to investigate tinnitus-related distress [18], and our study was similar in that patients were asked to use VAS to indicate the degree of tinnitus loudness on a scale from 0 to 10. The study showed a positive statistically significant correlation between THI and VAS scores before and after therapy, with the higher the degree of tinnitus distress, the higher the VAS score, which was another corroboration of previous studies. However, the correlation between HADS-A/D and VAS scores was poor, which is consistent with previous studies that tinnitus-related emotional disorders were independent of tinnitus loudness [19].

Due to the complex etiology and unclear mechanisms of tinnitus, there was still no specific effective treatment. The psychological model proposed by Taylor suggested that the overall annoyance of tinnitus was caused by the characteristics of the tinnitus and the psychological makeup of each patient. Several parts of the brain are supposed to involve in the representation of tinnitus and the response to it. Treatments could be focused on reducing tinnitus (e.g., medication) or reducing the response to it (e.g., counseling) [20]. Currently, the commonly used clinical therapies for subjective tinnitus include tinnitus masking therapy and TRT [21]. A recent study showed that sound therapy on its own, can help some tinnitus sufferers without any counseling [22]. Li et al. [23] studied frequency-based personalized music therapy by running a controlled trial and found a significant decrease in THI scores. The complex nature of tinnitus requires an attempt to create sound therapies individually tailored to each unique tinnitus condition. Sound therapy approach used in this study was a personalized web-based customized music therapy which was created by software using a computational model that combined each individual's auditory threshold and self-assessed tinnitus characteristics to predict changes in neural connectivity and activity. In terms of pathophysiology, the probable cause of tinnitus is abnormal excitability or central remodeling in the brainstem, subcortex, or cerebral cortex caused by damage to the peripheral auditory system [24], If model simulations indicate a decrease in neural activity that may be associated with tinnitus [25], music with altered spectral variation spectrum is added to the music therapy package for that patient which will gradually reverse the remolding or reduce the excitabiity of auditory cortical correlates of tinnitus through acoustic activation of putative neuroplasticity mechanisms. Music was chosen as the object rather than white noise or modulated sound because of its proven positive effect on cortical plasticity [26], which potentially increases patient compliance while providing a more pleasant listening experience.

Most importantly, our study was implemented based on a complete follow-up system. During treatment, regular telephone follow-ups are conducted, which can not only accurately record the time patients spent listening to music and remind them if the therapy time was insufficient, but also inform them of the therapy progress and results. If patients were found to have significant emotional fluctuating during treatment, face-to-face consultation was advised to relieve their symptoms through conservative treatments such as medication. A complete follow-up system was expected to reduce the therapy time, patients' stress, anxiety and loss of hope. Previous domestic and international clinical studies with small samples can provide one-on-one telephone follow-up of enrolled patients [20–23]. However, it seemed unlikely to carry out in clinical practice for a large sample of patients, as such a complete follow-up system is very important. Meanwhile, our previous study found that tinnitus patients not only need proper guidance and technical support, but also encouragement and counseling to persist, while most patients give up sound therapy once the impact of tinnitus on their lives is reduced. Therefore, our previous study showed that it was difficult to cure tinnitus out of hospital with acoustic therapy alone, but if combined with an effective follow-up system, the outcome will be greatly improved [27]. Nevertheless, our trial had a LTF rate of 3.58% after 3 months of treatment, and these patients were statistically mainly those who improved significantly or recovered after therapy. This LTF has a high probability of not affecting our statistical results, but it also indicates that our consultation and follow-up system needs to be optimized, as we recommend that music therapy be continued for at least three months.

With the development of clinical diagnosis and treatment, although the clinical efficacy of customized sounds for tinnitus have been confirmed, there is a lack of large-scale systematic research in this area, and the present prospective large sample study aims to assess the improvement of the results by using personalized music therapy. The results showed that THI scores decreased after therapy, and the degree of decrease correlated with the baseline values. Note that this overall THI reduction (16 points) is lower than that reported by María Cuesta [28], which was 23 points in 80 patients. The main reason could be that the current study included patients with THI from 4–100 points, whereas María Cuesta excluded patients with THI < 20. In addition, the data from a large sample of clinical studies are more reflective of the general population, and since the patients in this study came from all over the country, we assumed that the data we obtained are, to some extent, representative of the Chinese tinnitus population.

Studies have shown that tinnitus is often associated with significant emotional distress, particularly depression and anxiety, all of which contribute to decreased quality of life [29]. There are neurobiological relationships between tinnitus and anxiety and depressive disorders based on some overlapped brain networks [30], stemming primarily from the connection between limbic and auditory systems. Therefore, assessment of emotional disorders would be important for the assessment of tinnitus. The main outcome in assessment study showed that the prevalence of anxiety and depression in patients with chief complaints of tinnitus was 70.57% and 40.65%, respectively, and percentage of patients with anxiety or depression was up to 75.28% which was rather high, suggesting that anxiety and depression were comorbidities of tinnitus in China. Meanwhile, the different prevalence of anxiety and depression in tinnitus patients indicated that patients with chief complaints of tinnitus have more anxiety disorders than depression (*P* < 0.01). After music therapy, median of anxiety dropped by 2 points, while depression dropped by only 1 point, which suggested that music therapy was more effective in tinnitus-related anxiety than depression. Also, our study showed that if the efficacy of music therapy on anxiety and depression was assessed by tinnitus severity, after 3 months of therapy, in terms of anxiety, there were statistically significant differences in all groups (including catastrophic, severe, moderate, mild group) between pre- and post-therapy values, except for the mild group. While in terms of depression, only the catastrophic and moderate groups showed statistical differences (data not shown). This provides an important basis for our clinical treatment of tinnitus. The treatment of anxiety and depression includes not only music therapy, but also psycho-spiritual aspects for better results.

This study confirmed that the initial severity of tinnitus and VAS score were factors that influence the effectiveness of customized music therapy, and the treatment effect of patients with severe or catastrophic tinnitus (THI ≥ 58) was 3.537 times higher than that of patients with mild to moderate tinnitus, and the lower the subjective tinnitus loudness of patients, the better the therapy efficacy. This has an important role in guiding clinical treatment, and based on these results clinicians can give reasonable recommendations and strategies for tinnitus patients with different characteristics. Tinnitus duration is closely related to central remodeling, the longer the duration, the less likely the reversal of the remodeled central will occur and the less effective the music treatment will be. In addition, tinnitus related anxiety prior to therapy was also an influencing factor. This might because tinnitus patients with comorbid anxiety had higher compliance with therapy due to concerns about the possible adverse consequences of tinnitus. Other relevant control variables such as age, gender and frequency of tinnitus, laterality and whether one is in a depressive state have no effect on the treatment effect of customized sound. The fact that depression did not affect the therapy effect may be related to the negative emotions of distrust/suspicion to the treatment strategy, which play a key role in triggering negative physical and psychological changes in the body and has an impact on the tinnitus itself, thus creating a feedback loop that further aggravates the perception of tinnitus [30]. Therefore, maintaining a positive and optimistic attitude in dealing with tinnitus is a key factor in improving the symptoms of tinnitus patients.

This study has some limitations. First, this study was not a randomized double-blind controlled study which was unlikely to be implemented in clinical practice, either from a therapeutic or ethical point of view. Besides, it is also unknown whether this sound therapy only acts as a placebo as well. If possible, future studies need to consider inclusion of a comparison control group. Second, we did not stratify our analysis by the presence or absence of hearing loss which is considered the main risk factor to develop tinnitus. Future studies will focus on the response of tinnitus patients with different hearing levels to this customized music therapy. Third, longer follow-up duration is recommended to evaluate the lasting efficacy of therapy in the future studies.

## Conclusion

Our study is the first large sample-sized prospective study to evaluate the efficacy of personalized and customized music therapy combined with a well-designed follow-up system for Chinese tinnitus patients. The follow-up system is designed to encourage, supervise, record data and provide consultation for each patient individually. The results suggest that music therapy can reduce tinnitus distress and improve the symptoms of tinnitus comorbidities, including anxiety and depression. Baseline THI/VAS scores, duration of tinnitus and anxiety were the statistically significant factors affecting the efficacy of music therapy. Although there are some shortcomings, there is no doubt that this study could be the first step before this music therapy could be largely used in clinical setting.

## Data Availability

Not applicable.
